# Natural attack rate of influenza in unvaccinated children and adults: a meta-regression analysis

**DOI:** 10.1186/s12879-014-0670-5

**Published:** 2014-12-11

**Authors:** Kavisha Jayasundara, Charlene Soobiah, Edward Thommes, Andrea C Tricco, Ayman Chit

**Affiliations:** GSK Inc, 7333 Mississauga Road North, Mississauga, L5N 6L4 ON Canada; Li Ka Shing Knowledge Institute of St. Michael’s Hospital, 30 Bond Street, Toronto, M5B 1W8 ON Canada; Department of Mathematics & Statistics, University of Guelph, 50 Stone Road East, Guelph, N1G 2W1 ON Canada; Division of Epidemiology, Dalla Lana School of Public Health, University of Toronto, 155 College St, Toronto, M5T 3M7 ON Canada; Sanofi Pasteur, 1755 Steeles Avenue West, Toronto, M2R 3T4 ON Canada

**Keywords:** Influenza A, Influenza B, Natural attack rate, Healthy individuals, Systematic review

## Abstract

**Background:**

The natural (i.e. unvaccinated population) attack rate of an infectious disease is an important parameter required for understanding disease transmission. As such, it is an input parameter in infectious disease mathematical models. Influenza is an infectious disease that poses a major health concern worldwide and the natural attack rate of this disease is crucial in determining the effectiveness and cost-effectiveness of public health interventions and informing surveillance program design. We estimated age-stratified, strain-specific natural attack rates of laboratory-confirmed influenza in unvaccinated individuals.

**Methods:**

Utilizing an existing systematic review, we calculated the attack rates in the trial placebo arms using a random effects model and a meta-regression analysis (GSK study identifier: 117102).

**Results:**

This post-hoc analysis included 34 RCTs (Randomized Control Trials) contributing to 47 influenza seasons from 1970 to 2009. Meta-regression analyses showed that age and type of influenza were important covariates. The attack rates (95% CI (Confidence Interval)) in adults for all influenza, type A and type B were 3.50% (2.30%, 4.60%), 2.32% (1.47%, 3.17%) and 0.59% (0.28%, 0.91%) respectively. For children, they were 15.20% (11.40%, 18.90%), 12.27% (8.56%, 15.97%) and 5.50% (3.49%, 7.51%) respectively.

**Conclusions:**

This analysis demonstrated that unvaccinated children have considerably higher exposure risk than adults and influenza A can cause more disease than influenza B. Moreover, a higher ratio of influenza B:A in children than adults was observed. This study provides a new, stratified and up to-date natural attack rates that can be used in influenza infectious disease models and are consistent with previous published work in the field.

**Electronic supplementary material:**

The online version of this article (doi:10.1186/s12879-014-0670-5) contains supplementary material, which is available to authorized users.

## Background

Infectious diseases are a major cause of morbidity and mortality worldwide [1]. The main focus for research on infectious diseases is to improve disease control and ultimately eradicate the infection from the population [2]. Mathematical models of infectious diseases are important for studying the impact of various interventions on disease outcome. Such investigations allow the implementation of targeted control measures, optimizing the use of limited resources [2].

The validity of a model is highly dependent on the validity of the input parameters. These models require information on parameters describing the population under study, disease transmission as well as parameters describing the health outcomes of infection. Lack of reliable data complicates the development of models and new data needs to be collected or analyzed or the model itself needs to be fitted to available data to estimate input parameters [3].

Influenza is an infectious disease that poses a major health concern all over the world. The total economic burden of influenza in the United States has been estimated as $87.1 billion [4]. Developing mathematical models for influenza can be used to determine how to mitigate disease. The attack rate of the influenza virus is crucial for these models and a difficult parameter to obtain.

Generally, influenza epidemic infection rates are obtained from influenza-like illness (ILI), surveillance data and serological surveys [5]. In a static (e.g. decision tree) model, the attack rate is often used directly as an input parameter, whereas in a dynamic transmission model, it is used to calibrate, or calculate directly, the force of infection. Whether static or dynamic, it is most preferable to use the “natural attack rate”, that is, the attack rate within the unvaccinated portion of the population. This is a more “universal” quantity, since it is not as strongly dependent on the population vaccine uptake, though it is still coupled through herd immunity.

Turner *et al*. used age-specific attack rates of influenza in an economic evaluation based on rates derived from placebo-controlled trials of influenza vaccines and antivirals [6]. The incidence of symptomatic laboratory-confirmed influenza cases in each placebo arm was used to estimate the corresponding attack rates for each subgroup using a random effects model. Overall, the attack rate was 6.55% (range: 0 to 20%) for adults and 19.21% (range: 10 to 35%) for children. All trials except one were in the US and spanned eight influenza epidemics during the period 1984 through 1997-1998. These attack rates were not adjusted for confounders and also were not stratified by strain of influenza.

The objective of this study was to estimate the attack rate of influenza stratified by both age and influenza strain (A and B) for unvaccinated individuals measured by laboratory-confirmed PCR (Polymerase Chain Reaction) and/or culture.

## Methods

This study (GSK study identifier: 117102) is a post-hoc analysis of a published systematic review [7]. The full methods have been published [7],[8] and the protocol was registered in the PROSPERO database (CRD42012001926).

### Eligibility criteria

Healthy children, adults or the elderly were chosen as the population of interest. All vaccine types were included: trivalent influenza vaccines (TIV), live-attenuated influenza vaccines (LAIV), and bivalent vaccines. Randomized controlled trials (RCTs) or quasi-RCTs, written in English, comparing influenza vaccine with placebo and reporting data on the primary outcomes were included. The primary outcome used was laboratory-confirmed influenza by PCR and/or culture [7]. The secondary outcome included haemagluttination inhibition (HAI) assay alone or in combination with PCR and/or culture.

### Information sources and search

Medical subject headings (MeSH) and text words related to influenza vaccination were used in the literature search strategies. MEDLINE, EMBASE and Cochrane Central Register of Controlled Trials were searched by an experienced librarian. This was supplemented by searching for trial protocols through *meta*Register (http://www.controlled-trials.com/mrct/), which allows the searching of multiple trial protocol registries simultaneously. The reference lists of included studies and relevant reviews were scanned to ensure all studies were captured. Authors of studies were contacted for additional information, as necessary.

### Study selection process

Online proprietary systematic review software *Synthesi.SR* (http://knowledgetranslation.ca/sysrev/login.php), available through the Li Ka Shing Knowledge Institute of St. Michael’s hospital was used to screen citations and full-text articles that were identified through the literature search. After a team calibration exercise, the *Synthesi.SR* program was used to screen citations and full-text articles. Each citation and full-text article was screened by two reviewers independently.

### Data items

The study characteristics abstracted were as follows: year of conduct, sample size, country of conduct, setting, viral strain(s) detected (strain A or B), age of study population (mean and standard deviation), infection detection method (PCR, viral culture, HAI), number of confirmed influenza infections in the placebo arm and the total number of patients in the placebo arm.

### Synthesis of included studies

The data was first summarized descriptively. The natural attack rate was computed using the following formula: number of influenza cases/number of subjects in the placebo arm × 100%, as per the methods in Turner *et al*. [6]. The overall natural attack rate of influenza for all unvaccinated individuals was derived using a random effects model which allows for within and between study variance [6]. Separate analyses were conducted for children and adults (children: <18 years, Adults: ≥18 years), strain of influenza confirmed by laboratory testing (strain A or B), the type of test used for laboratory confirmation (PCR and/or culture vs. HAI testing alone or in combination with PCR or culture). Time trends for attack rates were observed through forest plots.

### Meta-regression analysis

Important covariates that would influence the natural attack rate of influenza were identified *a priori.* They were age group, geographical region where the study was conducted, type of laboratory-confirmed influenza detected (all influenza, strain A and strain B), type of test used to confirm infection (PCR and/or culture vs. HAI testing alone or in combination with PCR or culture) and the year of influenza season. Univariate analyses were conducted on these covariates to determine their statistical significance. Based on those results, meta-regression analysis was performed on statistically significant covariates.

It is not always preferred to run all possible covariates in a meta-regression model as it would reduce statistical power of the results and lead to larger confidence intervals. The total number of covariates was constrained so that there were enough studies per outcome [9]. Additionally, statistical heterogeneity was tested using the I^2^ statistic [10]. If high heterogeneity was observed, meta-regression analysis was conducted. All statistical analyses were conducted using *R version 2.1*.

## Results

**Figure 1:**
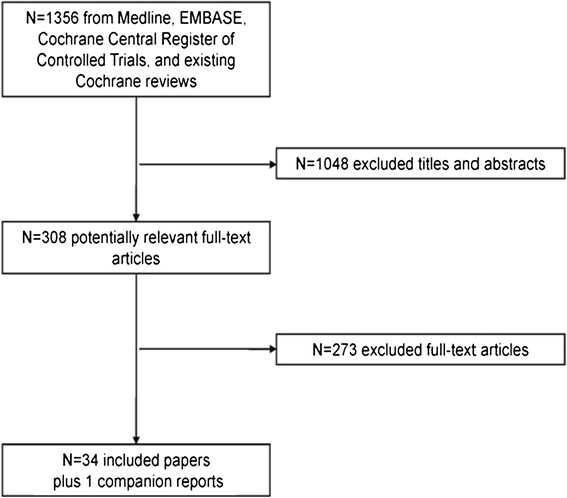
**Study flow chart.**

The systematic review informing this analysis yielded 1356 citations [7]. A total of 34 RCTs and 1 companion report fulfilled the inclusion criteria (Figure 1). These 34 trials contributed to 47 influenza seasons from 1970 to 2009 including 94,821 participants. Reasons for exclusion, study characteristics, and patient characteristics are reported in detail elsewhere [7]. The trials were conducted in North America (22/34), Europe (4/34), Australia (1/34) and a mixture of multi-site trials in North America, Europe, South America, Asia (7/34).

From the 47 influenza seasons identified, 37 reported on the primary outcome (laboratory confirmed influenza by PCR and/or culture) and all 47 reported on the secondary outcome (laboratory confirmed influenza by HAI assay alone or in combination with PCR and/or culture). From the 37 influenza seasons that reported on the primary outcome, 34 reported on influenza A and 35 reported on influenza B. From the 47 influenza seasons that reported on the secondary outcome, 41 reported on influenza A and 42 reported on influenza B. Attack rates were calculated for all age groups separated by type of influenza using a random effects model (Table 1). All results are reported in the following format: point estimate, 95% confidence interval [CI, (lower level, upper level)]. For the primary outcome, the overall attack rate was 7.86% (5.48%, 10.23%) for all influenza, 5.68% (3.71%, 7.65%) for strain A and 1.87% (1.02%, 2.72%) for strain B. For the secondary outcome, an attack rate of 9.84% (7.06%, 12.62%) was observed for all strains, 6.96% (4.49%, 9.42%) for strain A and 2.57% (1.45%, 3.70%) for strain B. The results of the random effects model also demonstrated high statistical heterogeneity the high I^2^ values.

**Table 1:** **Meta-analysis results comparing primary outcome (RT-PCR and/or culture) with secondary outcome (RT-PCR and/or culture and/or HAI assay) for all ages**

Type of influenza	# of influenza seasons	Sample size (placebo group)	# of influenza cases (placebo group)	Natural attack rate % (lower 95% CI, upper 95% CI)	I ^2^
**Primary outcome (PCR and/or viral culture)**					
All	37	39115	1774	7.86 (5.48, 10.23)	99.86%
Influenza A	34	38360	1303	5.68 (3.71, 7.65)	99.69%
Influenza B	35	38854	491	1.87 (1.02, 2.72)	99.87%
**Secondary outcome (PCR and/or viral culture and/or HAI assay)**					
All	47	41212	2055	9.84 (7.06, 12.62)	99.77%
Influenza A	41	40074	1505	6.96 (4.49, 9.42)	99.80%
Influenza B	42	40568	536	2.57 (1.45, 3.70)	99.93%

*Abbreviations:* *CI* confidence interval, *HAI* haemagglutination inhibition assay, *RT-PCR* reverse transcription polymerase chain reaction.

Meta-regression analysis was conducted to reduce the heterogeneity associated with the attack rate calculations. Age was considered an important covariate in natural attack rate. In order to confirm this with our data, a meta-regression analysis was conducted with age stratifications. With the available data from the studies from the systematic review, we were only able to separate ages into two categories: adults and children. Limited number of trials reported on the elderly population alone and most of them did not report the primary outcome. It was difficult to use geography as a covariate since many trials were multi-national in nature. Due to this limitation, we did not include geography as a covariate in the meta-regression.

Data was separated by primary and secondary outcome and type of influenza to determine whether adults and children experience different attack rates. The results of the meta-regression analysis are shown in Table 2. For all influenza, adults exhibit an attack rate of 4.4% and children experienced an attack rate that was 11% higher than adults. For influenza A, adults exhibited an attack rate of 3.4% and children experienced a higher attack rate than adults by 8.8%. For influenza B, adults showed an attack rate of 1.1% and children showed an attack rate that was 4.4% higher than that of adults. All analyses were statistically significant and suggested that a subgroup analysis by age group is required for the primary outcome for all influenza, influenza A and B.

**Table 2:** **Meta-regression for primary outcome**

	Estimate	Standard error	P-value
**Influenza A and B**			
(Intercept)	0.044	0.012	0.0012
Children vs. Adults	0.110	0.019	<0.0001
**Influenza A**			
(Intercept)	0.034	0.011	0.005
Children vs. Adults	0.088	0.018	<0.0001
**Influenza B**			
(Intercept)	0.011	0.0055	0.046
Children vs. Adults	0.044	0.009	<0.0001

When a similar meta-regression was conducted on the broader secondary outcome, higher attack rates were observed overall. Results can be seen in Table 3. Adults, on average, exhibited at attack rate of 5.8% for all influenza with children experiencing a rate that is 13.9% higher than that of adults. For influenza A, adults experienced an attack rate of 4.2% with children having an attack rate 7.9% higher than adults. Attack rate of influenza B was lower than that of influenza A, with adults exhibiting an attack rate of 1.4% and children having an attack rate that is 5.6% higher than that of adults. These analyses were all statistically significant. The trends of higher attack rates for children than adults, and higher attack rates for strain A than strain B were also present when analyzing the secondary outcome.

**Table 3:** **Meta-regression for secondary outcome**

	Estimate	Standard error	P-value
**Influenza A and B**			
(Intercept)	0.058	0.018	0.0024
Children vs. Adults	0.139	0.028	<0.0001
**Influenza A**			
(Intercept)	0.042	0.018	0.0225
Children vs. Adults	0.079	0.0279	0.0074
**Influenza B**			
(Intercept)	0.014	0.015	0.375
Children vs. Adults	0.056	0.024	0.025

Since we confirmed that age and type of influenza were important covariates for natural attack rates, we then conducted subgroup analysis by age group (adults and children) and type of influenza (all, type A and type B) for the primary outcome (Table 4). The overall attack rate for adults for all influenza was 3.50% (2.30%, 4.60%) and when separated by type, 2.32% (1.47%, 3.17%) for influenza A and 0.59% (0.28%, 0.91%) for influenza B. For children, the overall attack rate was 15.20% (11.40%, 18.90%) for all influenza. When stratified by type, 12.27% (8.56%, 15.97%) for influenza A and 5.50% (3.49%, 7.51%) for influenza B. Trials that reported outcomes specifically on adults and children were included in this analysis and trials that reported on mixed populations were excluded. Even with the sub group analysis by age and type of influenza we still observed high I^2^ values.

**Table 4:** **Meta-analysis on primary outcome for adults and children**

Type of influenza	# Influenza seasons	Sample size (placebo group)	# Influenza cases (placebo group)	Natural attack rate % (lower 95% CI, upper 95% CI)	I ^2^
**Adults**					
All	19	28748	627	3.50 (2.30, 4.60)	98.0%
Influenza A	19	28748	482	2.32 (1.47, 3.17)	97.90%
Influenza B	19	28748	150	0.59 (0.28, 0.91)	98.11%
**Children**					
All	13	6187	1040	15.20 (11.40, 18.90)	94.0%
Influenza A	10	5432	714	12.27 (8.56, 15.97)	94.59%
Influenza B	11	5926	341	5.50 (3.49, 7.51)	94.69%

*Abbreviations:* *CI* confidence interval.

**Figure 2:**
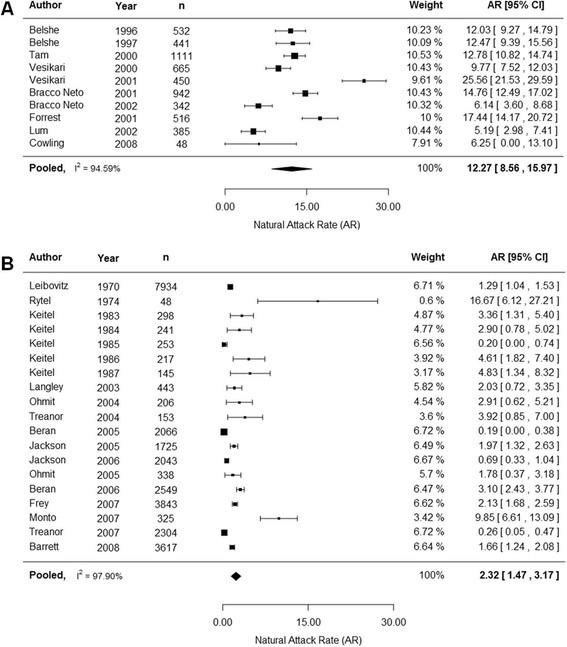
**Natural attack rate of influenza A in children (A) and adults (B) based on the primary outcome.** Children were defined as <18 years and adults as ≥18 years. Abbreviations: CI confidence interval.

**Figure 3:**
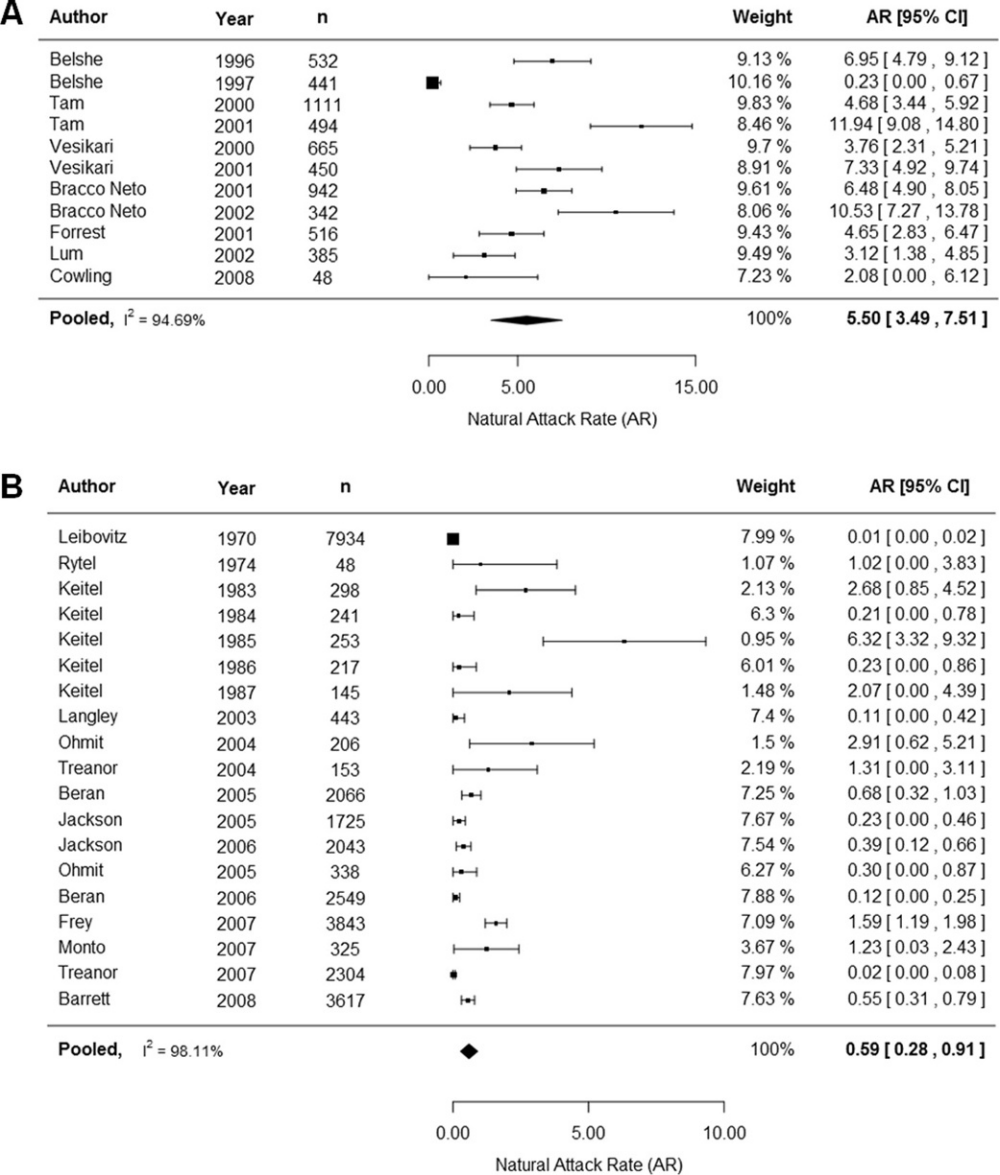
**Natural attack rate of influenza B in children (A) and adults (B) based on the primary outcome.** Children were defined as <18 years and adults as ≥18years. Abbreviations: CI confidence interval.

The forest plot for influenza A infections in children and adults for the primary outcome can be seen in Figure 2A and B respectively. The forest plot for influenza B for children and adults can be seen in Figure 3A and B respectively. Generally, no time trends were observed in the attack rate for adults or children for each type of influenza.

## Discussion

This study estimated the natural attack rate of influenza for unvaccinated children and adults using a post-hoc analysis of a previously conducted systematic review. This is the best method to obtain such attack rates since currently existing methods to obtain this data have a number of limitations. First, estimating attack rates from surveillance data provides attack rates as an aggregate of vaccinated and unvaccinated individuals and not attack rates that are specific to unvaccinated individuals that are presented here. Second, extrapolating from surveillance data also necessitates estimating rates of primary care consultations among influenza cases [5]. Third, even if there is an active surveillance system looking for influenza, surveillance systems will be limited by under-reporting bias [11]. Finally, it’s difficult to determine the size of the population under surveillance which makes estimating rates of infection difficult as there is uncertainty around the denominator of the ratio [11].

Although yearly influenza epidemics can seriously affect all age groups, children younger than two years of age and adults over 65 exhibit higher rates of disease [12]. Further, contact pattern studies suggest that children are more vulnerable to infection due to their relatively more frequent and longer intimate contacts with members of their age group [13]. Therefore, age is believed to be important in the natural attack rate of influenza. Geography dictates contact patterns and seasonality which is known to be a driver in severity of influenza [14]. Outbreaks of seasonal influenza follow largely predictable seasonal patterns and in temperate climate zones seasonal influenza epidemic lasts from six to ten weeks and in tropical zones seasonal patterns are less pronounced with more than one peak of infection [15]. Thus, geography is also an important consideration with regards to natural attack rate. With respect to the two main types of influenza in circulation, influenza A contributes to greater annual epidemics and infrequent yet more-devastating pandemics than influenza B [16]. Therefore, it’s important to distinguish between these two types of influenza types. As the sensitivity of various laboratory methods for detecting influenza can yield different results, this was also included as an important covariate. Since yearly differences in influenza are seen from season to season, the year of study conduct was also considered an important covariate. Therefore, age group, geographical region, type of laboratory-confirmed influenza detected, type of test used to confirm detection and the year of influenza season were all considered important covariates of natural attack rate in our study.

Meta-regression analyses conducted here showed that age group and type of influenza are important covariates in natural attack rates. For adults, an attack rate of 3.50% (2.30%, 4.60%) was observed for all influenza strains. When stratified by type, an attack rate of 2.32% (1.47%, 3.17%) was observed for influenza A and a rate of 0.59% (0.28%, 0.91%) was observed for strain B. For children, the overall attack rate was 15.20% (11.40%, 18.90%) and when stratified, a rate of 12.27% (8.56%, 15.97%) was seen for strain A and 5.50% (3.49%, 7.51%) was seen for strain B. The weighted sum of the stratified rates may not add up to the aggregate rates as some studies reported results for both strains of influenza. Including HAI testing increased the attack rate for all age groups and all types of influenza. No general trends with time were observed for children and adults for the primary outcome.

This study provides new, up to-date estimates of the attack rate of influenza in children and adults separated by type of influenza. Generally, our overall attack rates for adults and children were lower than the attack rates observed by Turner *et al*. [6]. The Turner analysis included trials covering only 8 influenza seasons from 1984 to 1998 while our analysis covered 47 influenza seasons from 1970 to 2009. The primary outcome of our studies was PCR and/or culture but the studies used in the Turner analysis did not include PCR as a method of detection. PCR is considered the most reliable diagnostic test for clinical practice with a higher accuracy of detection [17]. Using culture as a method of detection may miss influenza cases and the use of HAI testing may lead to biased results [17]. This may explain why the attack rates observed in the Turner analysis are higher than the results we have obtained (6.55% for adults versus 3.57% and 19.21% for children versus 14.22%). Our secondary outcome, which included HAI testing, also showed an increase in attack rates for all age groups and types of influenza which supports the notion that higher attack rates are observed when HAI testing is used as a method of detection. Furthermore, the definition of children used by our study and by the Turner study is quite different. In our study, children were classified as <18 years while the Turner classified them as <12 years. This may also attribute to the differences seen in attack rates when comparing between studies. Influenza B is most prominent among older children and young adults [18]. In line with this, our attack rates for children exhibited a higher B:A ratio than for adults. The B:A attack rate ratio for children was calculated to be 0.45 while the ratio for adults was 0.25. This further exemplifies that using this method to calculate the natural attack rate does in fact reflect what is observed in real life settings for seasonal influenza [18]. The strengths of our analysis over the Turner analysis include the use of many trials covering more influenza seasons, using a more accurate method of detection (RT-PCR (Reverse Transcription Polymerase Chain Reaction) and/or culture), adjusting for confounders that affect the natural attack rate and providing strain-specific natural attack rates for influenza.

Like any meta-analysis, our study has several limitations. First, although we identified three RCTs reporting data in the senior population, we were not able to derive an attack rate for this population. Two out of the three studies did not report on our primary outcome of interest and the third study reported an attack rate of 0%. Second, we faced an inability to calculate geography-specific attack rates. Although it was decided *a priori* that geography is an important covariate, the data did not support such an analysis. Although most of the trials were conducted in North America, newer trials were multi-centre in nature spanning over more than one continent, making a geography-specific analysis difficult. Third, the attack rates that are calculated here are attenuated by community herd effects through vaccinated people in the population. For countries with a low background vaccination rate, natural attack rates might be higher than what’s reported here. As most of the studies in this analysis were conducted in North America, the attack rates obtained here would reflect the effects of herd immunity observe through targeted vaccine coverage programs that are used in this region. Furthermore, the attack rates observed in this study are laboratory confirmed. A study by Glatman-Freedman *et al*. have shown that in general, laboratory confirmed attack rates are higher than clinical attack rates which is an important consideration when using these rates as model inputs [19]. Another limitation of this study is that H1N1 and H3N2 strains were agglomerated as strain A for analysis. The attack rates for these two types for strain A are different from each other and thus lead to different health outcomes. Also, the severity of the influenza strains are known to be in the order of H3N2 > B > H1N1 [18]. Therefore, one must be cautious when applying the agglomerated attack rate presented here for strain A to reflect H3N2 or H1N1 specifically. Although we identified one study that focused on the H1N1 pandemic flu, Talaat *et al.* [20], it reported on adults and elderly population together and therefore was not included in the meta-regression of attack rate for adults. Lastly, as this study is based on the results of an already conducted systematic review, it is inherently limited by the results of the systematic review. The systematic review only focused on RCTs of vaccines and not antivirals (as was done for the Turner *et al*. analysis). If the focus of the systematic review was expanded to include antivirals, then a large sample size would have been available for the attack rate calculations and perhaps lead to reduced uncertainty of the estimates that were derived from the analysis.

Influenza models are important tools in public health decision-making, and natural attack rate of influenza is a vital ingredient for such models, as either a direct input parameter or a calibration target. It is imperative that robust data are used to inform these models so that they can better predict disease transmission and also resource allocation that is required to reduce the burden of illness. The sophistication of mathematical models of disease transmission has increased in the last decade [21],[22]; accordingly, the need for valid, precise and accurate model inputs, such as natural attack rate, is greater than ever. The over- or under-estimation of these parameters can have significant impact on estimating the burden of illness and can introduce uncertainty in public health decision making.

## Conclusions

In this study, we were able to determine important covariates for natural attack rate of influenza and calculate age-specific and strain-specific attack rates that would serve as essential input parameters to an infectious disease model for influenza. We believe that the use of robust and accurate input parameters are key to reducing uncertainty associated with infectious disease models and therefore would make them more appealing in public health decision making settings.

## Authors’ original submitted files for images

Below are the links to the authors’ original submitted files for images. Authors’ original file for figure 1 Authors’ original file for figure 2 Authors’ original file for figure 3

## Abbreviations

PCR: Polymerase chain reaction
CI: Confidence interval
ILI: Influenza-like illness
TIV: Trivalent influenza vaccines
LAIV: Live-attenuated influenza vaccines
RCT: Randomized controlled trial
HAI assay: Haemagluttination inhibition assay
MeSH: Medical subject headings
RT-PCR: Reverse transcription polymerase chain reaction
