# Supplementary figures and images for: Natural attack rate of influenza in unvaccinated children and adults: a meta-regression analysis
Source: BMC Infect Dis. 2014 Dec 11;14:670. doi: 10.1186/s12879-014-0670-5 (PMC4272519; doi:10.1186/s12879-014-0670-5)

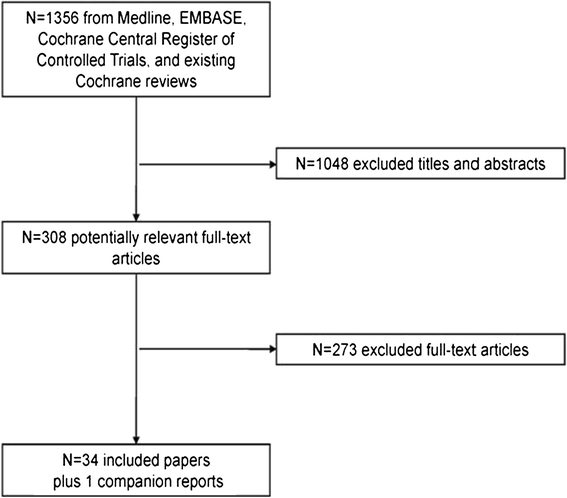

Supplement: Supplementary file 1 — Authors’ original file for figure 1 [file 12879_2014_670_MOESM1_ESM.gif]

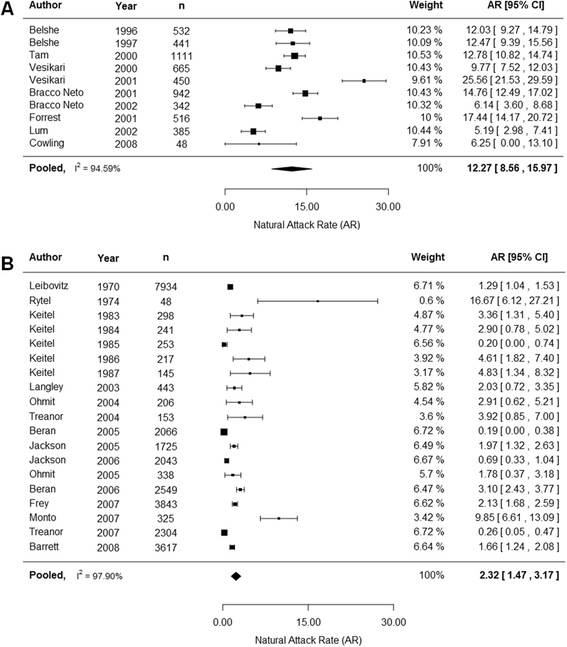

Supplement: Supplementary file 2 — Authors’ original file for figure 2 [file 12879_2014_670_MOESM2_ESM.gif]

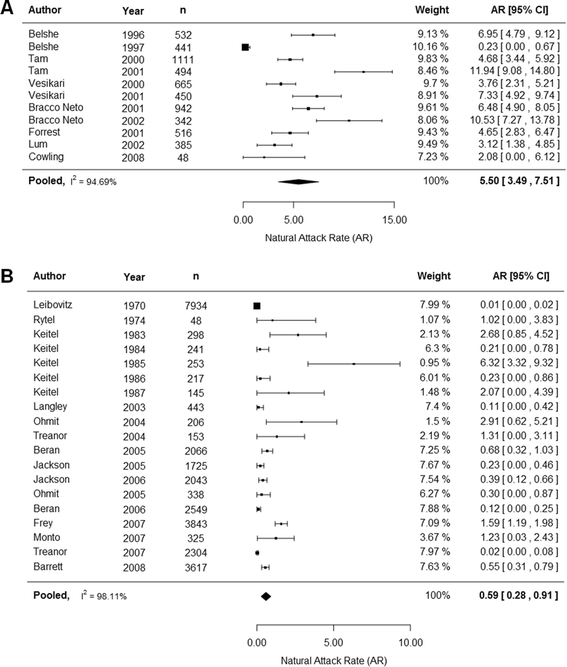

Supplement: Supplementary file 3 — Authors’ original file for figure 3 [file 12879_2014_670_MOESM3_ESM.gif]
